# Evaluation of Modified Categorical Data Fuzzy Clustering Algorithm on the Wisconsin Breast Cancer Dataset

**DOI:** 10.1155/2016/4273813

**Published:** 2016-02-24

**Authors:** Amir Ahmad

**Affiliations:** Faculty of Computing and Information Technology in Rabigh, King Abdulaziz University, P.O. Box 344, Rabigh 21911, Saudi Arabia

## Abstract

The early diagnosis of breast cancer is an important step in a fight against the disease. Machine learning techniques have shown promise in improving our understanding of the disease. As medical datasets consist of data points which cannot be precisely assigned to a class, fuzzy methods have been useful for studying of these datasets. Sometimes breast cancer datasets are described by categorical features. Many fuzzy clustering algorithms have been developed for categorical datasets. However, in most of these methods Hamming distance is used to define the distance between the two categorical feature values. In this paper, we use a probabilistic distance measure for the distance computation among a pair of categorical feature values. Experiments demonstrate that the distance measure performs better than Hamming distance for Wisconsin breast cancer data.

## 1. Introduction

Breast cancer is the most common form of cancer amongst women [1]. Early and accurate detection of breast cancer is the key to the long survival of patients [1]. Machine learning techniques are being used to improve diagnostic capability for breast cancer [2–4]. Wisconsin breast cancer dataset has been a popular dataset in machine learning community [5]. Various classification techniques such as techniques like decision trees [6], support vector machines [7], and fuzzy-genetic algorithm [8] have been used to study this dataset. In medical datasets, sometimes it is difficult to put some data points in one of the groups. Fuzzy methods are better equipped to handle these kinds of datasets [9–11].

Clustering divides the data points into different groups (clusters) depending upon a similarity measure [12]. The data points in a group (cluster) are similar whereas data points in different groups (clusters) are dissimilar. Clustering algorithms can be divided into two groups [12, 13]: hard clustering algorithms and fuzzy clustering algorithms. In hard clustering, a data point can have a membership to a cluster. However, in fuzzy clustering, a data point has memberships to all the clusters.

*K*-means algorithm [14] is very popular hard clustering algorithm because of its linear complexity. *K*-means clustering algorithm is an iterative algorithm which computes the mean of each feature of data points presented in a cluster. This makes the algorithm inappropriate for the datasets that have categorical features. Huang [15] extends the *K*-mean algorithm for the datasets having categorical features. Instead of mean, mode is used to represent a cluster. Hamming distance is used to calculate the membership of a data point. In Hamming distance if the feature values are same for two data points the distance is taken as 0; otherwise the distance is taken as 1.

Hierarchical clustering algorithms [12] can be applied for categorical datasets; however they have high computation complexity. This makes them less useful for large datasets.

Fuzzy clustering has shown great promise in understanding medical datasets [10, 11]. It has been shown that the fuzzy clustering can be used to improve the classification performance of various classifiers for diagnosis of breast cancer [16]. Fuzzy *c*-mean (FCM) [17, 18] is one of the most popular clustering techniques. Original FCM clustering technique can only handle numeric features. Using the methodology of FCM, fuzzy *K*-mode algorithm [19] is proposed for categorical datasets. This method use Hamming distance and hard cluster centres. Kim et al. [20] propose a fuzzy clustering algorithm that uses fuzzy cluster centres. This algorithm performs better than fuzzy *K*-mode algorithm [20].

Most of fuzzy clustering algorithms for categorical datasets use Hamming distance. However, Lee and Pedrycz [21] show that the simple matching similarity like Hamming distance cannot capture the correct similarities among categorical feature values; hence an appropriate distance measure should be used to improve the performance of fuzzy clustering algorithm with fuzzy cluster centres.

Various dissimilarity measures have been proposed for categorical feature values [23]. Ahmad and Dey [22] present a dissimilarity measure for categorical features. Ahmad and Dey [22] show that *K*-mode clustering algorithm can be improved with this dissimilarity measure. Ahmad and Dey [24] use this distance measure to propose a clustering algorithm for datasets having numerical and categorical features. Ahmad and Dey [25] also suggest a subspace clustering algorithm with this dissimilarity measure. Motivated by the success of the dissimilarity measure for clustering categorical data, Ji et al. [26] use the distance measure for fuzzy clustering of mixed datasets. Ahmad and Dey [27] presented a fuzzy clustering method that uses a distance measure that calculates distances for each iteration.

Wisconsin breast cancer dataset has been studied extensively in machine learning field [25, 30–32]. Each feature of Wisconsin breast dataset has ten categories (1 to 10). It has been a popular dataset for analysing clustering algorithms for categorical datasets [25, 30–32]. In this paper, we show the application of the clustering algorithm proposed by Ji et al. [26] for Wisconsin breast cancer dataset. This way we will show the applicability of the distance measure proposed by Ahmad and Dey [22] for the analysis of categorical breast cancer dataset.

This paper has the following organization. We will discuss fuzzy *c*-mean clustering algorithm in Section 2.  Section 3 reviews the method that computes the distance between two categorical feature values.  Section 4 discusses the method to compute the fuzzy centroid for categorical datasets and the distance between a data point and a cluster centre [26]. Experimental results are presented in Section 5.  Section 6 has conclusion and future work.

## 2. Fuzzy *c*-Mean Clustering Algorithm

Fuzzy *c*-mean (FCM) [17, 18] is a popular clustering algorithm. In this section, we will discuss FCM.

The following information is given:

(i) A dataset is with *n* data points.
(ii) Each data point is defined by *s* features.
(iii) The desired number of clusters is *K*.
(iv) Fuzzy membership matrix *U* = (*u*_*ij*_)_*n*×*K*_.

FCM clusters a set of *n* data points, *X* = {*X*_1_, *X*_2_,…, *X*_*n*_}, into *K* clusters, where *X*_*i*_ = {*x*_*i*,1_,…, *x*_*i*,*s*_} is the *i*th data point for *i* = 1,…, *n*.

FCM compute the cluster centres *v*_*j*_  (*j* = 1,2,…, *K*), where *v*_*j*_ = {*v*_*j*1_, *v*_*j*2_,…, *v*_*js*_}, and the fuzzy membership matrix *U*. It is done by minimizing an objective function, *J*, presented below iteratively:

(1) $$\begin{array}{c} J=\overset{K}{\underset{j=1}{\sum }}\overset{n}{\underset{i=1}{\sum }}{\left(\;u_{ij}\;\right)}^{m}d_{ij} \end{array}$$

(*m* is used as defined real number which controls the fuzziness)

(2) $$\begin{array}{c} \text{subject to}\;\;\overset{K}{\underset{j=1}{\sum }}u_{ij}=1,\;i=1,2,\ldots ,n, \end{array}$$

where *d*_*ij*_ is the distance between data point *X*_*i*_ and cluster centre *v*_*j*_.

For numeric data, *v*_*j*_ and *u*_*ij*_ are computed as follows:

(3) vjp=∑i=1nuijmXip∑i=1nuijm,uij=1∑k=1Kdij/dkj1/m−1.

The steps for FCM based algorithm are presented as follows.

**Step 1 .**

Select a stopping value *ε*. Initialize the fuzzy membership matrix* U*. It is done by creating *n* × *K* random numbers; these numbers are in the interval [0,1].

 
*do*

**Step 2 .**

Compute cluster centres.

**Step 3 .**

Compute distances from centres and use these distances for updating fuzzy membership matrix *U*.

**Step 4 .**

Calculate the objective function *J*.

 
*While* (the difference between two subsequent computed values of *J* is more than the given stopping value *ε*).

## 3. The Distance between Two Categorical Feature Values

Ahmad and Dey [22] propose an algorithm to calculate the distance between two categorical feature values in an unsupervised framework. Unlike Hamming distance, this distance measure does not take binary measure for the distance between two categorical values. The distance is calculated by computing the cooccurrence of the feature values (for which the distance is calculated) with feature values of other features.

The distance between categorical feature values *x* and *y* of feature *A*_*i*_ against the feature *A*_*j*_, for a subset *w* of feature *A*_*j*_ values, is defined as follows:

(4) $$\begin{array}{c} \delta_{w}^{ij}\left(\;x,y\;\right)=p\left(\;\frac{w}{x}\;\right)+p\left(\;\sim \frac{w}{y}\;\right)-1. \end{array}$$

The distance *δ* ^*ij*^(*x*, *y*) between the feature values *x* and *y* for *A*_*i*_ against feature *A*_*j*_ is presented by *δ* ^*ij*^(*x*, *y*) and is defined by *δ* ^*ij*^(*x*, *y*) = *p*(*ω*/*x*) + *p*(~*ω*/*y*) − 1, where *ω* is the subset *w* of feature values of *A*_*j*_ that maximizes the quantity (*p*(*w*/*x*) + *p*(~*w*/*y*)). To compute the distance between *x* and *y*, we compute the distances between *x* and *y* against every other feature. The average distance is taken as the distance, *δ*(*x*, *y*), between *x* and *y* in the dataset. Distances between every pair of feature values are employed to calculate the distance between a data point and a cluster centre.

## 4. Modified Centre and the Distance from the Modified Centre

For categorical datasets, the mode is used to calculate the centre of clusters [19]. However, taking only one feature value to represent a cluster centre does not capture the cluster centre well; hence loss of information takes place. Ji et al. [26] use the fuzzy centroid [20] concept with distance measure suggested by Ahmad and Dey [22] for fuzzy clustering of categorical datasets.

The fuzzy centroid for a cluster, *C*, for a categorical dataset is defined as
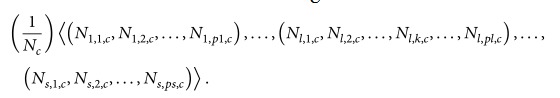
(5)Assume that *l*th feature has *pl* different values.

Thus,

(6) $$\begin{array}{c} N_{c}=\overset{n}{\underset{i=1}{\sum }}{\left(\;u_{ij}\;\right)}^{m}\;\left(\text{where }j\text{th cluster is }C\right), \end{array}$$

where *N*_*l*,*k*,*c*_ is the association of value *A*_*l*,*k*_ (*k*th feature value for the *l*th feature) with cluster *C*:

(7) $$\begin{array}{c} N_{l,k,c}=\overset{n}{\underset{i=1}{\sum }}L\left(\;X_{il}=A_{l,k}\;\right){\left(\;u_{ij}\;\right)}^{m}\;\left(j\text{th cluster is }C\right), \end{array}$$

where  *L*(*X*_*il*_ = *A*_*l*,*k*_)

 = 1 for a data point *X*_*i*_ having *l*th feature value = *A*_*l*,*k*_,
 = 0 for a data point *X*_*i*_ having *l*th feature value ≠*A*_*l*,*k*_.

The distance between a data point having *l*th categorical feature value *Z* in the *l*th dimension and the centre of cluster *C* is defined as

(8) ΩZ,C=Nl,1,cNc∗δZ,Al,1+Nl,2,cNc∗δZ,Al,2+⋯+Nl,t,cNc∗δZ,Al,t+⋯+Nl,pl,cNc∗δZ,Al,pl,

where *A*_*l*,*t*_ is the *t*th feature value of *l*th categorical feature.

*δ*(*x*, *y*) is calculated by the method discussed in Section 3. For dataset having *s* features, the distance is calculated for each feature value of the data point and the summation of these distances is the distance between the data point and the centre. In FCM, the distances between data points and cluster centres are used to calculate fuzzy membership matrix. Hence, this distance measure will be employed to compute the fuzzy membership matrix.

The cluster centre definition and distances between cluster centre and data points discussed in this section can be used with FCM algorithm discussed in Section 2 to create fuzzy clustering algorithm for categorical datasets [26]. The steps of fuzzy clustering algorithm for categorical data are as follows.

**Step 1 .**

Select a stopping value *ε*. Initialize the fuzzy membership matrix *U*. It is done by creating *n* × *K* random numbers; these numbers are in the interval [0,1].

 
*do*

**Step 2 .**

Compute cluster centres by using (5).

**Step 3 .**

Compute distances from centres by using (8). Hamming distance/distances discussed in Section 3 will be used in this step. Use these distances for updating fuzzy membership matrix* U*.

**Step 4 .**

Calculate the objective function *J*.

 
*While* (the difference between two subsequent computed values of* J* is more than the given stopping value *ε*).

## 5. Results and Discussion

The experiments were carried out on Wisconsin breast cancer data. This dataset has 699 data points. Each data point is represented by 9 features. 16 data points have missing values. Missing feature values were replaced by the mode of that feature. The information about these features is given in Table 1. These are two groups in this dataset: benign and malignant. Benign group has 458 data points whereas malignant group has 241 data points. Each feature has categories (0–10). We ran fuzzy clustering with fuzzy centroid with Hamming distance and the distance measure proposed by Ahmad and Dey [22] to see how the incorporation of the distance measure affects the quality of the clustering.

 
*Clustering error* = the number of data points not in desired clusters/the number of data points.

To assess the quality of clustering, it is assumed that a preclassified dataset is provided and the “overlap” between an achieved clustering and the ground truth classification is measured. Experiments were carried out at different values of *m*: 1.1, 1.5, and 1.9. The random initialization was used for both clustering algorithms. Clustering algorithms were run 100 times in each setting (different *m* values) and average results are presented in Table 2. We also presented the performance of various clustering algorithms on Wisconsin breast cancer dataset. We performed the experiments for fuzzy *K*-modes clustering algorithm. The average result of 10 runs with *m* = 1.1 is presented. Other clustering results are taken from [30]. Results are presented in Table 3. Confusion matrices for different setups are presented in Tables 4 [Table tab5] [Table tab6] [Table tab7] [Table tab8]–9.

Clustering results suggest that for all values of *m* the fuzzy clustering algorithm with Ahmad and Dey [22] distance measure performed better; for example, for *m* = 1.1, the average clustering error for the proposed algorithm was 5.0%, whereas the average clustering error with Hamming distance was 10.4%. This shows that the application of the distance measure improved the clustering results. Table 3 suggests that the fuzzy clustering algorithm with Ahmad and Dey [22] distance measure performed better than other clustering algorithms.

**Figure 1:**
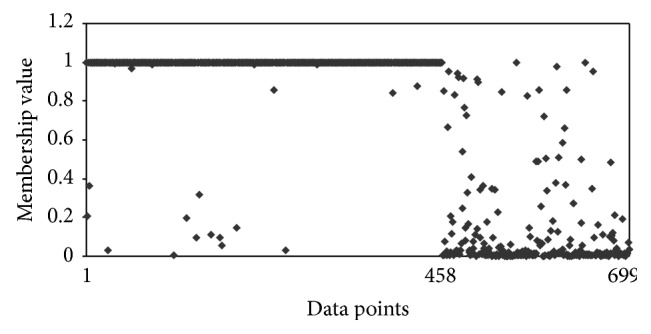
Membership for different data points for the benign cluster by using Ahmad and Dey [22] distance measure for *m* = 1.1. The first 458 data points are benign and next 241 data points are malignant.

**Figure 2:**
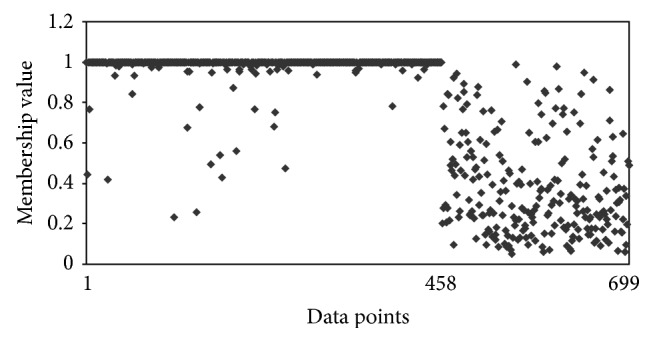
Membership for different data points for the benign cluster by using the Hamming distance for *m* = 1.1. The first 458 data points are benign and next 241 data points are malignant.

**Figure 3:**
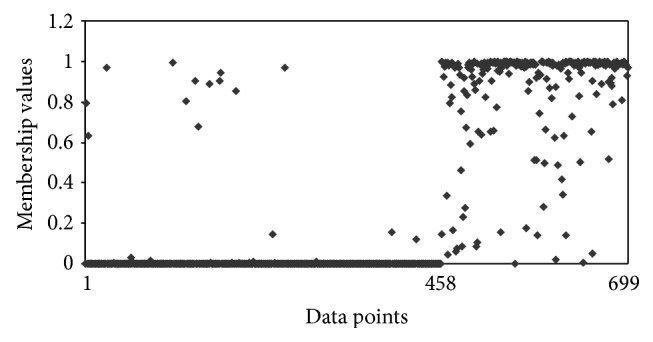
Membership for different data points for the malignant cluster by using Ahmad and Dey [22] distance measure for *m* = 1.1. The first 458 data points are benign and next 241 data points are malignant.

**Figure 4:**
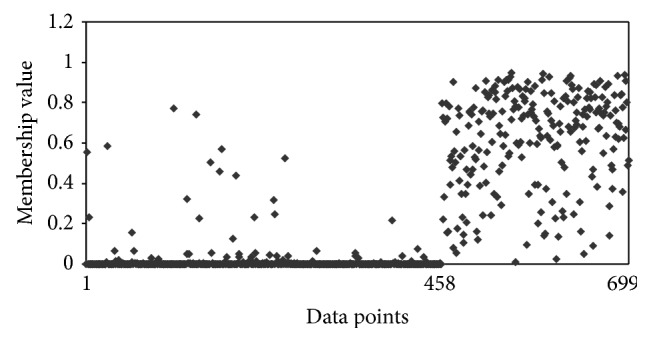
Membership for different data points for the malignant cluster by using the Hamming distance for *m* = 1.1. The first 458 data points are benign and next 241 data points are malignant.

The other interesting observation is that, with Hamming distance, the clustering algorithm was putting malignant data points in benign clusters. In other words, it had difficulty in assigning malignant data points correctly, whereas the clustering algorithm with Ahmad and Dey [22] distance measure had better assignment of malignant data points. To understand this point more, we compared the membership of different data points for these two algorithms. Figures 1 and 2 show the membership of different data points for benign cluster. It shows that with Hamming distance even the malignant data points have high memberships for benign cluster. However, with the distance measure proposed by Ahmad and Dey [22], we have better membership relationship. Figures 3 and 4 show the membership of different data points for malignant cluster. This suggests that with Hamming distance membership values of benign data points are low; however, membership values of malignant data points are not as high as that with the proposed algorithm. These observations demonstrate that, with the distance measure proposed by Ahmad and Dey [22], better membership values were achieved.

## 6. Conclusion and Future Work

Early and correct detection is the key for the cure of breast cancer. Machine learning techniques are important diagnostic tools for breast cancer. Fuzzy clustering algorithms have shown great promise in analysis of breast cancer. Wisconsin breast cancer dataset has been treated as a categorical dataset in different studies because its features have categories (1–10). Ahmad and Dey [22] suggested a distance measure that has been successfully used in many clustering algorithms for categorical datasets. We used this distance measure for fuzzy clustering of Wisconsin breast cancer dataset. Our results suggest that we got better results as compared to the fuzzy clustering algorithm with Hamming distance. Experiment results also suggest that the membership values achieved by the distance measure proposed by Ahmad and Dey [22] better matched the given information. In future, we will apply this distance measure to other medical datasets. Various other fuzzy clustering algorithms for categorical datasets have been suggested [33–35]; in future, we will study the applicability of the distance measure proposed by Ahmad and Dey [22] for these algorithms. A comparative study of other distance measures will also be carried out [36]. The cluster centre initialization is a problem as different random initialization leads to different clustering results [37]. In future, we will apply different cluster centre initialization methods to overcome this problem.

## Figures and Tables

**Table 1:** Information about features.

Feature number	Feature
1	Clump thickness
2	Uniformity of cell size
3	Uniformity of cell shape
4	Marginal adhesion
5	Single epithelial cell size
6	Bare nuclei
7	Bland chromatin
8	Normal nucleoli
9	Mitoses

**Table 2:** Average clustering results with standard deviation, in brackets, for Wisconsin breast cancer dataset. For all values of *m*, Ahmad and Dey [22] distance measure performed better.

The value of *m* (the measure of fuzziness)	Clustering error with Ahmad and Dey [22] distance measure algorithm in %	Clustering error with Hamming distance in %
1.1	5.0 (0.3)	10.4 (0.8)
1.5	4.7 (0.4)	10.3 (0.7)
1.9	5.0 (0.6)	9.7 (1.1)

**Table 3:** Comparative clustering results for Wisconsin breast cancer dataset.

Clustering algorithm	Clustering error in %
Fuzzy clustering with Ahmad and Dey [22] distance measure for *m* = 1.1	5.0
Fuzzy clustering with Hamming distance for *m* = 1.1	10.4
Fuzzy *K*-modes clustering for *m* = 1.1	13.9
Squeezer [28]	13.2
GAClust [29]	18.4
CcdByEnsemble [30]	15.0

**Table 4:** Confusion matrix for the average clustering result with Ahmad and Dey [22] distance measure, for *m* = 1.1.

	Cluster 1	Cluster 2
Benign	11	447
Malignant	217	24

**Table 5 tab5:** Confusion matrix for the average clustering result with Hamming distance, for *m* = 1.1.

	Cluster 1	Cluster 2
Benign	9	449
Malignant	177	64

**Table 6 tab6:** Confusion matrix for the average clustering result with Ahmad and Dey [22] distance measure, for *m* = 1.5.

	Cluster 1	Cluster 2
Benign	12	446
Malignant	220	21

**Table 7 tab7:** Confusion matrix for the average clustering result with Hamming distance, for *m* = 1.5.

	Cluster 1	Cluster 2
Benign	14	444
Malignant	183	58

**Table 8 tab8:** Confusion matrix for the average clustering result with Ahmad and Dey [22] distance measure, for *m* = 1.9.

	Cluster 1	Cluster 2
Benign	12	446
Malignant	218	23

**Table 9:** Confusion matrix for the average clustering result with Hamming distance, for *m* = 1.9.

	Cluster 1	Cluster 2
Benign	9	449
Malignant	182	59
